# Ground-up-top down: a mixed method action research study aimed at normalising research in practice for nurses and midwives

**DOI:** 10.1186/s12912-017-0249-8

**Published:** 2017-09-12

**Authors:** Vicki Parker, Gena Lieschke, Michelle Giles

**Affiliations:** 1 School of Health, University of New England, Armidale, 2351 NSW Australia; 2 Centre for Nursing and Midwifery Research, Hunter New England Local Health District, Newcastle, NSW 2300 Australia

**Keywords:** Action research, Practice-based research, Capacity building, Normalisation process theory (NPT)

## Abstract

**Background:**

Improving health, patient and system outcomes through a practice-based research agenda requires infrastructural supports, leadership and capacity building approaches, at both the individual and organisational levels. Embedding research as normal nursing and midwifery practice requires a flexible approach that is responsive to the diverse clinical contexts within which care is delivered and the variable research skills and interest of clinicians. This paper reports the study protocol for research being undertaken in a Local Health District (LHD) in New South Wales (NSW) Australia. The study aims to evaluate existing nursing and midwifery research activity, culture, capacity and capability across the LHD. This information, in addition to input from key stakeholders will be used to develop a responsive, productive and sustainable research capacity building framework aimed at enculturating practice-based research activities within and across diverse clinical settings of the LHD.

**Methods:**

A three-phased, sequential mixed-methods action research design underpinned by Normalization Process Theory (NPT).

Participants will be nursing and midwifery clinicians and managers across rural and metropolitan services. A combination of survey, focus group, individual interviews and peer supported action-learning groups will be used to gather data. Quantitative data will be analysed using descriptive statistics, correlation and regression, together with thematic analysis of qualitative data to produce an integrated report.

**Discussion:**

Understanding the current research activity and capacity of nurses and midwives, together with organisational supports and culture is essential to developing a productive and sustainable research environment. However, knowledge alone will not bring about change. This study will move beyond description of barriers to research participation for nurses and midwives and the promulgation of various capacity building frameworks to employ a theory driven action-oriented approach to normalisation of nursing and midwifery research practice. In doing so, our aim is to make possible the utilisation, generation and translation of practice based research that informs improved patient and service delivery outcomes.

**Electronic supplementary material:**

The online version of this article (10.1186/s12912-017-0249-8) contains supplementary material, which is available to authorized users.

## Background

Recognition of the dynamic interplay between research and practice is essential to improving the quality of nursing practice. Research yields new evidence that is vital for improving health outcomes for patients and families. Given that nurses and midwives are uniquely placed in relation to health service delivery, patients, their families and carers, these professionals have an important contribution to make in improving patient and health outcomes via practice-based research [1–4]. Nurses’ and midwives’ knowledge and attitudes toward research have been examined extensively over time, as have barriers to nurses and midwives undertaking research [5–8]. However, little attention has been paid to examining the contextual dynamics that impact on the ability to establish a research rich environment and an active and productive clinician research workforce.

Review of the literature highlights the importance of capacity building with appropriate support, at all levels; working with individuals, teams and organisational systems and processes [3]. Further, fostering environments that are conducive to continuous service improvement through research has been shown to be fundamental to embedding research within clinical service delivery [3]. In spite of this extensive work, it remains that few nurses undertake research, and very little truly practice-based research is conducted either by, or in collaboration with clinicians. Notwithstanding the need to establish clinical research career pathways accompanied by formal education, working on the ground in the real world of practice is critical to making research possible and meaningful for nurses and midwives.

This paper outlines a protocol for a study designed to examine and build the research activity, capacity, capability and culture for nurses and midwives and to embed research as a normal legitimised element of clinical practice.

Research capacity has been described as a critical element required to advance nursing and midwifery research and development, and foundational to these professions providing clinical practice excellence [9–11]. Finch [4] defines research capacity as “enhancing the ability within a discipline or professional group to undertake high quality research” (p. 427), and Murphy et al. [12] describes capacity building as the “individual and organisational developments which lead to the greater ability to access, conduct and apply useful research” (p.14). Condell and Begley’s [13] description of the concept - ‘research capacity building’ provides additional clarity. The authors offer the following, not as a definitive definition, but rather a description of the term as it is applied in the literature and derived from their concept analysis; That is,

“research capacity building implies, a funded, dynamic intervention operationalised through a range of foci and levels to augment the ability to carry out research or achieve objectives in the field of research over the long term, with aspects of social change as an ultimate outcome” (p. 273).

Condell and Begley’s [13] definition has been adopted for the purpose of this study.

Various research capacity building frameworks (RCBF) utilised to enable and support a culture of critical enquiry whilst simultaneously developing individual, team and/or organisational research capacity and capability have been reported in the literature. Descriptive accounts of these approaches range from; providing clinicians with targeted and structured research training and skills development opportunities and initiatives [14, 15], coordinating research activity around the deployment of key individuals, teams and units that are responsible for mentoring and leading clinicians through small local, practice-based research projects [9, 16–18], and whole of service/organisation models that aim to increase research activity and capacity across communities or groups of health care clinicians with a shared goal for driving a collective research agenda, usually within specialist multidisciplinary clinical contexts [12, 19–21].

These descriptive accounts have more recently given way to aggregated interpretations of what has been identified as the essential elements of a productive and sustainable approach to building research capacity within the disciplines of nursing and midwifery [22, 23]. A narrative review undertaken by O’Byrne and Smith [22] of publications from 1999 to 2010 identified three dominant models utilised for research capacity building in nursing and midwifery. The authors described these models as;

*“*the *practice based model *where research implementation and evaluation are prioritized; *the experiential learning model* whereby opportunities to develop research skills are provided through being supported to participate in collaborative research projects; and *the facilitative model* that integrates the above models to enable a broader approach to co-ordinating research activity, support and education across centres, units and networks in order to target the wider workforce” (p.1367).

Whilst O’Byrne and Smith acknowledge their review was limited by inconsistent definitions of capacity building across studies, together with a lack of evaluation studies with clearly identified outcome measures, it represents a comprehensive presé of activity and focus to date, and has identified a number of critical factors that enable the development of research capacity.

O’Byrne and Smith [22] identify collaboration, mentorship and availability of resources as enablers to research capacity. They also noted corroboration within the literature regarding the requirement for a cohesive plan with strong leadership and investment from managers. These findings support Cooke’s [24] six principles of research capacity building described in her multilevel (individual, team, organisational and supra-organisational) framework for planning and evaluating research capacity building in health care;

1. developing skills and confidence
2. developing linkages and partnerships
3. ensuring the research is close to practice
4. developing appropriate dissemination
5. investment in infrastructure
6. building elements of sustainability and continuity ([24], p.3).

Consistent with Cookes’ principles, Scala et al.’s [23] integrative review identified, “access to infrastructure, leadership support, strategic priorities and relevant interest, educational tactics and leveraging established networks and resources” (p. 428) as key themes associated with successfully engaging clinician nurses in research.

Whilst these critical elements for success have been reported consistently, few studies evaluating capacity building initiatives have been published. Such studies are necessary in order to provide evidence and guidance in the development of models that can be applied and adapted across a range of contexts. This study will examine the dynamic relationship between research and nursing practice in a large health district in New South Wales, Australia. It adopts a participatory action approach to implementation and evaluation of research capacity building in clinical practice.

## Methods

### Aim

The aim of the study is to identify existing Nursing and Midwifery research activity, capacity, capability and culture within the local health district (LHD) and to embed research as a normal component of nursing practice within clinical practice contexts.

The specific objectives of the study are to:

- Understand the current views and attitudes of nurses and midwives in relation to practice-based research.
- Identify current research expertise, practice and participation of nurses and midwives.
- Understand barriers and enablers of research participation for nurses and midwives.
- Gain consensus about what processes, networks and supports are required to normalise nursing and midwifery research practice.
- Understand how context influences nursing and midwifery research culture.
- Determine the critical success elements of a productive, sustainable and transferable research integration implementation model.

### Design

**Fig. 1:**
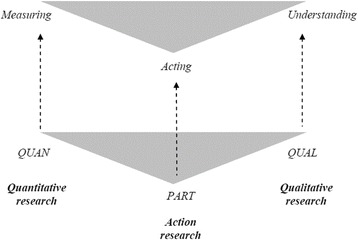
Methodological approaches, methods and aims. Baseline (and simple) model. Figure 1 taken from Martí [26] (with author and publisher permission) originally published in Action Research

A three-phased, sequential mixed methods action research study design will be utilised. Mixed methods action research takes a participatory, performative focus, integrating quantitative and qualitative methods with an action research methodological approach [25]. Martís’ [26] diagrammatic representation below (Fig. 1) highlights how combining methods in this ways allows for the integration of measurement and understanding to inform collective action.

Integration of methods will be achieved by taking the evidence derived from quantitative data to participants for reflection and action in focus groups and in action learning sets where it will be used to inform decision making and action to embed research within clinical contexts through cycles of observation, reflection, planning and action. Greenhalgh et al. [27], support the utilisation of Participatory Action Research (PAR) within implementation research, acknowledging the reciprocal interactions between context and program success. This approach will engage nurses and midwives across the LHD working in diverse clinical settings who have varying degrees of research experience, peer and organisational support and accountabilities associated with research.

However, mixed methods action research is not without challenges. Navigation of integration throughout the cycles of the action research process will be particularly challenging, as will including stakeholders as co-researchers and designing workable and accessible methods [25]. Adjunctive utilisation of normalization process theory (NPT) will help mitigate these challenges.

### Normalization Process Theory

Normalization Process Theory (NPT), first described by May in 2007 [28] and extended by May, Johnson and Finch in 2015 [29], will be used to guide the implementation and embedding of change into the complex and dynamic ‘real life’ nursing and midwifery practice contexts.

May [28] describes the journey of implementing and embedding interventions into complex environments as “trajectories of contingency”. That is, the processes by which agents negotiate and reconcile the “contending, conflicting and contingent and sometimes turbulent patterns of social action and relations, and their distribution across social time and space” (p. 27). NPT has four general assumptions;

1. Innovations become embedded in practice as the result of agents working individually and collectively to enact them; that

- Embedding of innovations is accomplished through generative mechanisms that take the form of agentic contributions by individuals and groups in processes of;
- Coherence; cognitive participation; collective action; and reflexive monitoring. Mechanisms that are shaped by;

3. Organising structures and social norms that specify the rules and roles that frame action, and the group processes and interactional conventions through which action is accomplished, and that;
4. The reproduction of an innovation requires continued investments by agents in ensembles of action that carry forward in time and space. (p. 27)

The theory offers a framework within which to identify and understand the contribution that individual and collective groups of nurses and midwives make (or do not make), within and across dynamic and complex contexts as they negotiate the normative and relational environment in which they work. Work is required to accommodate research activity as an embedded and integrated feature of routine nursing and midwifery practice. Additionally, the complex and interdependent relationship between how nurses and midwives successfully (or unsuccessfully) embed and integrate research into routine practice, and what facilitative or obstructive contingencies operate to influence these outcomes will be illuminated. These findings will be useful in informing RCBFs that are responsive to nurses’ and midwives’ diverse and dynamic needs, but also in providing a robust process and outcome evaluation related to these interventions. Table 1 outlines how NPT will inform action learning sessions in the final phase of the study.

**Table 1:** Application of normalising process theory

	Questions	Desired outcome
Coherence	- What is the nursing and midwifery research capacity? - How is this evident in our organisation? - What do we want to achieve and how can we go about it? - What benefits will accrue in building research capacity amongst nurses and midwives?	- Shared goals - Role identification - Finding value
Cognitive participation	- How do we get buy in from stakeholders? - How do we reconstruct research as legitimate work for N&M in clinical practice environments? - How do we sustain our efforts? - How do we garner sponsorship, commitment, and resources from senior and executive ﻿level managers﻿?.	- Working together - Reorganisation of work patterns - Legitimisation – defining actions - Staying on the case
Collective action	- How can we work together to achieve shared goals? - Taking responsibility and being accountable. - Who will lead initiatives?	- Operational work - Interaction - Relational integration - Skill set workability (Who gets to do what) - Allocation of resources
Reflexive monitoring	- What are we learning? - How can our learning inform our thinking and actions?	- Appraisal - Redefinition - Refinement

### A phased approach

**Fig. 2:**
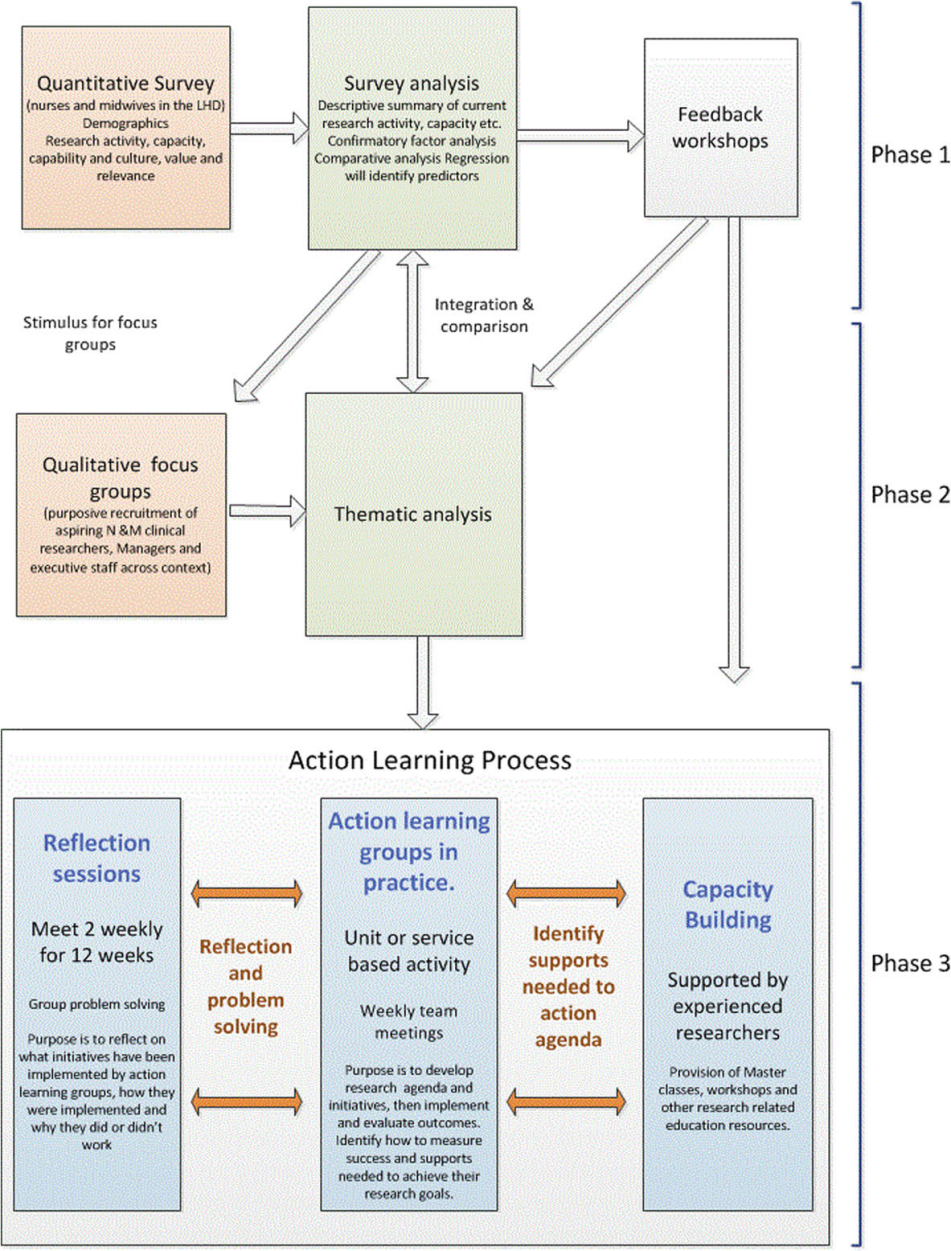
Study design

The study has three sequential phases outlined in Fig. 2.

#### Phase one

A LHD wide cross-sectional online survey of all nurses and midwives. Undertaking this initial scoping exercise will be important in providing base-line information about the;

- nature of nursing and midwifery research across the LHD
- existing research capacity within the disciplines
- prevailing nursing and midwifery research culture
- perceived barriers confronting nurses and midwives wanting to undertake practice-based research, and
- existing enablers within the LHD that support nurses and midwives undertaking practice-based research within the LHD.

### Survey development

The survey was developed based on review of existing and modification of previously validated instruments (Additional file 1). The survey consists of 31 questions related to research activity, capacity, capability and culture, as well as previously identified barriers and enablers to research activity practice and participation in health. The constructs of the survey are detailed in Table 2. The survey also aims to provide demographic information and an overview of current Nursing and Midwifery research activity, individual skills, intentions across the LHD.

**Table 2:** Survey constructs

	Definition	Measurement tool
Individual domain		
Perceived individual research intention	Individual’s intent to engage with research activities and opportunities in order to inform their practice.	Research and Development Culture Index (R & D Culture Index) [33]
Perceived individual research capacity	Individual skill level across a variety of research related activities from finding the literature through to dissemination of findings.	Research and Development Culture Index (R & D Culture Index) [33] Research and Capacity Culture Tool (RCC Tool) [8]
Perceived research relevance	Importance individual places on research for practice improvement and significance in daily work, relevance to profession and relevance to education.	Nursing Research Questionnaire (NRQ) [6]
Perceived research value	Value and impact of research in practice and on their profession.	Nursing Research Questionnaire (NRQ) [6]
Perceived translation of research into practice	Explores whether research is collaborative between clinicians and researchers, is directed by strategic priorities, improves patient and organisational outcomes through sustained practice change and used to evaluate interventions.	
Organisational domain		
Perceived organisational support	Degree of organisational support and opportunity for, and application of research in your team or service.	Research and Capacity Culture Tool (RCC Tool) [8] Queensland Health Practitioner Research Capacity Survey [34]
Perceived organisational culture and capability	Degree of research related resources, planning, leadership, opportunities, consumer involvement, and quality monitoring and expert advice.	Queensland Health Practitioner Research Capacity Survey [34]

Permission was given by all authors to use part or all of their previously validated instruments.

### Survey sample group

The desired sample size with a power calculation that demonstrates a representative sample with a confidence level of 95% is 366. There are 8500 nurses and midwives employed across the LHD where the study will be undertaken. To ensure the sample will be selected correctly and the response adequate, the online survey link will be distributed to all nurses and midwives via their employment email address. Targeting all nurses and midwives will ensure a more representative sample and mitigate a possible low response rate.

### Survey analysis

Quantitative analysis will include a comprehensive descriptive summary to produce a demographic and geographic composite profile of nurse and midwife respondents.

Confirmatory factor analysis will be conducted on the scales from the previously validated tools that make up the survey using a variety of correlational analysis to examine the inter-relationships amongst multiple variables [30]. Cronbach alpha testing will be conducted on all scales and sub-scales used in the survey to measure the internal consistency of the subscales. Internal consistency describes the extent to which all the items in a test measure the same concept or construct and hence it is connected to the inter-relatedness of the items within the test with a value of 1 indicating that all items are measuring exactly the same latent variable [31].

The Translational research factor is not a previously validated instrument, but was designed by the research team. Cronbach alphas (to assess internal consistency) will be calculated on the seven items comprising this scale.

Comparative analysis using Mann Whitney U test will identify differences between groups. Logistic and linear regressions will be conducted on selected response outcomes to identify associations. Univariate regression will be conducted for each outcome and each covariate separately. Covariates with a *p*-value <0.20 and a relevant effect size in the univariate analysis will be considered for inclusion in the multivariable model for each outcome.

#### Phase two

Following feedback sessions reporting the survey findings**,** focus group interviews will be held with key stakeholders. The findings from the phase one survey will be used as stimuli to develop questions to generate focus group discussion as demonstrated in Fig. 2.

Focus group interviews will also be held across the District in both metropolitan and rural locations. Stakeholders invited to attend the focus group interviews will be purposively recruited as a consequence of their role, responsibilities, or experience related to research activity within the District (nurse and midwife senior clinicians, senior managers, researchers and nurse and midwife educators). Purposive recruitment of the above mentioned stakeholder groups will be via an invitation sent through work emails with information outlining the study and participant involvement required. Focus group discussions will be facilitated by the research investigators. Audio recorded focus group sessions will be transcribed and data analysed using thematic analysis informed by the NPT framework and interpretative description.

#### Phase three

The third phase aims to encourage and support nurses and midwives to engage in research capacity building processes. Several Action Learning Groups (ALG), consisting of senior nurse and midwife managers, clinical nurse consultants (CNCs) and nurse practitioners (NPs). Each of these groups will be supported and mentored by an experienced researcher who will work with the team for a period of 16 weeks to:

- Plan, develop, implement and evaluate a research conducive environment, with an established research agenda and research activity, within their respective units or departments
- Identify the support required to action their research agendas.
- The ALG groups will come together at fortnightly intervals in action learning sessions facilitated by experienced researchers. The purpose of the sessions is to discuss plans, progress, problems and issues arising. The experienced researcher will provide facilitation, mentorship, guidance and advice on all aspects of the research process. It is anticipated that each group will develop skills, knowledge and confidence in conducting practiced based research, with a view to embedding research as a normalised feature within clinical practice environments.

Data collection during this phase of the study will include notes from team meetings, and ward or unit specific data, that will inform the nature of the agenda and data related to identified outcome measures that will indicate success. Each of the fortnightly sessions will be digitally recorded with members’ consent, and will be transcribed for thematic and interpretative description analysis.

Groups will be requested to complete a journal across the 16 week timeframe that is structured around the four general assumptions within the NPT framework as outlined in Table 1. The multiple datasets will be analysed to determine achievement of desired outcomes based on the NPT framework to assess for example, whether participants have been able to successfully develop shared goals, work collectively and embed research activity into their clinical practice environments.

PAR is particularly important in achieving the study’s aim of supporting and enabling nurses and midwives to integrate research into clinical practice. Having an appreciation of the diverse personal and organisational cultural beliefs, along with an understanding of how political and system imperatives influence these care contexts will provide important insights into how practice-based research might successfully be improved across these diverse clinical contexts. In this way PAR enables solutions to be developed in response to context, and in doing so increases the likelihood that these transformations in practice-based research will be successful. Hence the approach is directed toward real-life rather than abstract situations and supports the concept that changing practice is a social process [32].

### Ethical consideration

The local Research Ethics and Governance Unit has granted ethics approval for the study (Reference number 15/12/16/5.09). All potential survey, focus-group and action learning group participants will be provided with details of the purpose of the study and their participation in each phase and be required to give informed consent. As a consequence of the group dynamic inherent within focus groups and action learning group discussions, care will be taken to remind participants that their respective anonymities can only be protected if all individuals agree to treat the information shared within the group, confidential to the group.

## Discussion

Increasingly, nurses and midwives are expected to drive improved patient outcomes through practice-based research. This protocol describes a novel approach to engaging nurses and midwives in practice based research. Using an action learning approach to capacity building at individual, team and organisation levels it aims to transcend barriers to research participation described in the literature and to align capacity building strategies and research activity with the goals and characteristics of varied contexts. It will also provide baseline data drawn from phases 1 and 2 to measure improvement over time. Using NPT to guide inquiry and action emphasises participation and shared goals as pillars of sustained change. In this way it is anticipated that the study will help to build a research culture and normalise research as integral to nursing and midwifery practice. It will also provide guidance to future initiatives through the development of robust evaluation measures and processes.

### Limitations of the study

Success of the study may be limited by the inability to engage and sustain the involvement of key stakeholders across the final two phases of the study and varying degrees of organisational support. Working with small numbers of groups in phase 3 will limit the generabilisability to other contexts, however allows a deeper engagement and the opportunity to identify a robust model of implementation.

## Additional file

Additional file 1: Survey tool. Survey used for phase one of study. (PDF 960 kb)

## Abbreviations

ALG: Action Learning Group
CNC: Clinical Nurse Consultant
LHD: Local Health District
NP: Nurse Practitioner
NPT: Normalization Process Theory
NSW: New South Wales
PAR: Participatory Action Research
RCBF: Research capacity building framework
