# Supplementary material for: Ground-up-top down: a mixed method action research study aimed at normalising research in practice for nurses and midwives
Source: BMC Nurs. 2017 Sep 12;16:52. doi: 10.1186/s12912-017-0249-8 (PMC5596929; doi:10.1186/s12912-017-0249-8)
Supplement: Additional file 1: — Survey tool. Survey used for phase one of study. (PDF 960 kb) [file 12912_2017_249_MOESM1_ESM.pdf]

Hunter New England Local Health District Nursing & Midwifery Research Survey 2015

1. About you.

The following questions relate to you, your educational qualifications and your employment status.

1. I am currently employed within HNELHD as a: (Please choose only one)

- ◯ RN
- ◯ RM
- ◯ RN and RM
- ◯ EN
- ◯ EEN
- ◯ CNS 1
- ◯ CNS 2
- ◯ CMS 1
- ◯ CMS 2
- ◯ CNC 1
- ◯ CNC 2
- ◯ CNC 3
- ◯ CMC 1
- ◯ CMC 2
- ◯ CMC 3
- ◯ NP
- ◯ Transitional NP
- ◯ CNE
- ◯ CME
- ◯ NE
- ◯ ME
- ◯ NUM
- ◯ MUM
- ◯ NM
- ◯ other

Specify other

[ ]

2. What is your current age in years?

[ ]

3. What is your gender?

- ◯ Female
- ◯ Male

4. How long have you been employed in HNELHD?

- ◯ < 1 years
- ◯ 1 to 3 years
- ◯ 3 to 5 years
- ◯ 5 to 10 years
- ◯ > 10 years

5. How long have you been in your current role?

- ◯ < 1 years
- ◯ 1 to 3 years
- ◯ 3 to 5 years
- ◯ 5 to 10 years
- ◯ > 10 years

6. Are you employed?

- ◯ Permanent full time (35 hours or more per week)
- ◯ Permanent part time (<35 hours per week)
- ◯ Temporary full time
- ◯ Temporary part time
- ◯ Casual
- ◯ Other

[ ]

7. Where is your current position *mainly* located?

- ◯ Rural location
- ◯ Metropolitan location
- ◯ Remote location
- ◯ Other (please specify)

[ ]

8. What category of health care do you *predominantly* work in?

- ◯ Prevention, Promotion and Protection
- ◯ Primary and community
- ◯ Ambulatory care
- ◯ Emergency care
- ◯ Acute care
- ◯ Rehabilitation and extended care
- ◯ Other (please specify)

[ ]

9. What is your *main* specialty area of practice?

- ◯ Midwifery
- ◯ Mixed medical and surgical
- ◯ Medical
- ◯ Surgical
- ◯ Critical care
- ◯ Emergency care
- ◯ Perioperative
- ◯ Paediatric
- ◯ Oncology / Haematology
- ◯ Aged care
- ◯ Palliative care
- ◯ Rehabilitation care
- ◯ Mental Health
- ◯ Family and Child Health
- ◯ Community Health
- ◯ Other (please specify)

[ ]

10. Please specify your *highest* health related educational qualifications and any study you are *currently undertaking* or *intend to undertake* in the next 2 years.

	Highest health related qualification	Currently undertaking further studies	Planning to undertake in the next 2 years
Not currently undertaking any studies	◯	◯	◯
Certificate	◯	◯	◯
Diploma	◯	◯	◯
Bachelor Degree	◯	◯	◯
Graduate Certificate	◯	◯	◯
Graduate Diploma	◯	◯	◯
Masters - Course work	◯	◯	◯
Masters - Research	◯	◯	◯
PhD	◯	◯	◯
Professional Doctorate	◯	◯	◯

Other (please specify)

[ ]

11. What research training courses have you undertaken? (choose as many as apply)

- [ ] Workshops
- [ ] Short Courses
- [ ] Rural Research Capacity Building Program
- [ ] None

Other (please specify)

12. If you are not already involved in research, would you like to become involved ?

- ( ) Yes
- ( ) No
- ( ) Not applicable

If Yes, In what areas would you like to conduct research?

Hunter New England Local Health District Nursing & Midwifery Research Survey 2015

2. Research Experience, Activity and Skill.

The following questions relate to your research experience(s), activity and skill.

1. Are you currently, or have you previously been involved in research within this LHD in the past 5 years?

- [ ] Yes
- [ ] No

2. How many research projects have you been involved with in the past 5 years in this LHD?

- ○ 1
- ○ 2
- ○ 3
- ○ 4
- ○ 5
- ○ more than 5
- ○ If more than 5, please specify number.

[ ]

3. Is the research you have been involved in been led by? (choose as many as apply)

- ☐ Nurses
- ☐ Midwives
- ☐ Medical Officers
- ☐ Allied Health
- ☐ Not sure

Other (please specify)

[ ]

4. What type of research have you been involved in? (choose as many as apply)

- ☐ Epidemiological studies
- ☐ Clinical Trials
- ☐ Qualitative studies
- ☐ Quantitative studies
- ☐ Mixed Method studies
- ☐ Not sure

Other (please specify)

[ ]

5. What is / was your role title in the research project/s (choose as many as apply)

- [ ] Project lead investigator
- [ ] Co-investigator
- [ ] Trial coordinator
- [ ] Research assistant
- [ ] Research manager
- [ ] Student
- [ ] Research Higher Degree Supervisor
- [ ] Mentor
- [ ] Academic advisor

Other (specify)

6. What were your main responsibilities in the research project/s? (choose as many as apply)

- [ ] Coordination of a project
- [ ] Definition of the area / topic of research
- [ ] Review of the literature
- [ ] Preparation of research protocol
- [ ] Preparation of grant application
- [ ] Study Design
- [ ] Preparation of ethics applications
- [ ] Recruitment of participants
- [ ] Providing intervention
- [ ] Data collection
- [ ] Data management
- [ ] Data analysis
- [ ] Writing up the research report / manuscript preparation
- [ ] Other (please specify)

7. Have any of the research projects you were involved in developed as a result of any of the following? (choose as many as apply)

- [ ] Continuous Practice Improvement / Quality
- [ ] Clinical audit activities
- [ ] Clinical Leadership Program
- [ ] Essentials of care
- [ ] Excellence
- [ ] None of the above
- [ ] Other (please specify)

8. Has the research you have been involved in changed policy and / or practice in any way?

- ○ Yes
- ○ No

If yes, please describe how the research findings have impacted on policy and practice.

9. Were any of the projects you were involved in funded or provided with resources or other supports ?

- ○ Yes
- ○ No
- ○ If Yes, please specify what type of funding, resources and supports?

10. How often have you undertaken the following in the past 3 years?

Presented research project findings at a unit level (in-service or grand rounds).

Presented research project findings at a District level.

Presented research project findings at a State or National level.

Presented research project findings at an international level.

Published project findings in a peer reviewed journal.

11. Please rate your level of agreement with the following statements?

	Strongly disagree 1	Disagree 2	Agree 3	Strongly agree 4
I understand research terminology	○	○	○	○
I feel confident about using research in my practice	○	○	○	○
I know how practice is influenced by research	○	○	○	○
I have the skills to use the library and learning facilities within the organisation or university	○	○	○	○
I would like to learn more about research activity	○	○	○	○
I would like more opportunity to share practice development ideas and research information	○	○	○	○
I am keen to use research in practice	○	○	○	○

12. Please rate your own level of skill for each of the following aspects of research by choosing a score from 1-10 (1 = no skill and 10 = highest possible skill)

	1	2	3	4	5	6	7	8	9	10
1. Finding relevant literature	◯	◯	◯	◯	◯	◯	◯	◯	◯	◯
2. Critically reviewing the literature	◯	◯	◯	◯	◯	◯	◯	◯	◯	◯
3. Using a computer referencing system (ie Endnote)	◯	◯	◯	◯	◯	◯	◯	◯	◯	◯
4. Writing a research protocol	◯	◯	◯	◯	◯	◯	◯	◯	◯	◯
5. Applying for research funding	◯	◯	◯	◯	◯	◯	◯	◯	◯	◯
6. Submitting an ethics application	◯	◯	◯	◯	◯	◯	◯	◯	◯	◯
7. Designing questionnaires	◯	◯	◯	◯	◯	◯	◯	◯	◯	◯
8. Recruiting study participants	◯	◯	◯	◯	◯	◯	◯	◯	◯	◯
9. Collecting data (Surveys, interviews)	◯	◯	◯	◯	◯	◯	◯	◯	◯	◯
10. Using computer data management systems	◯	◯	◯	◯	◯	◯	◯	◯	◯	◯
11. Analysing qualitative data	◯	◯	◯	◯	◯	◯	◯	◯	◯	◯
12. Analysing quantitative data	◯	◯	◯	◯	◯	◯	◯	◯	◯	◯
13. Writing a research report	◯	◯	◯	◯	◯	◯	◯	◯	◯	◯
14. Writing for publication in peer reviewed journals	◯	◯	◯	◯	◯	◯	◯	◯	◯	◯
15. Providing advice to less experienced researchers	◯	◯	◯	◯	◯	◯	◯	◯	◯	◯

Hunter New England Local Health District Nursing & Midwifery Research Survey 2015

3. Research Culture

The following questions relate to your attitudes towards research and your perception of the research culture within the unit or service in which you work.

1. Please rate your level of agreement with the following statements as they apply within your service / unit.

	Not at all 1	Infrequently 2	Sometimes 3	Frequently 4	Always 5
1. Research activities are mostly interdisciplinary in nature	○	○	○	○	○
2. Research activities are mainly done collaboratively between researchers and clinicians	○	○	○	○	○
3. Research activities are mostly directed by service strategic priorities	○	○	○	○	○
4. Research findings have improved patient and organisational outcome	○	○	○	○	○
5. Research findings have resulted in sustainable practice change	○	○	○	○	○
6. Research activities are well supported by management	○	○	○	○	○
7. Research is used to evaluate the impact of interventions on patient outcomes	○	○	○	○	○

2. Please rate your level of agreement with each of the following statements?

	1 Strongly disagree	2 Disagree	3 Agree	4 Strongly agree
1. I find that most reports of clinical research are too complex to understand	○	○	○	○
2. Most nurses / midwives are competent to undertake research with support	○	○	○	○
3. Most nurses / midwives are aware of relevant research findings	○	○	○	○
4. I feel that my manager encourages me to develop an interest in research	○	○	○	○
5. I feel that clinical research complicates the daily work	○	○	○	○
6. I value when some of my peers do research work in nursing / midwifery	○	○	○	○
7. We do not need any researchers in nursing / midwifery to develop our practice	○	○	○	○
8. Nurses / midwives are too busy delivering care to spend time reading research	○	○	○	○
9. I would be involved in research activities if the time was provided for me	○	○	○	○
10. Nurses / midwives are not in need of knowledge based on research as much as doctors are	○	○	○	○
11. Research is a specialist activity that is relevant to only a few nurses / midwives working in the clinical areas	○	○	○	○
12. Research is only relevant to nurse / midwife education, not to Nursing / Midwifery practice	○	○	○	○
13. Most clinical nurses / midwives are not interested in implementing research findings	○	○	○	○
14. Nursing / Midwifery should become a research based profession	○	○	○	○
15. Research findings have little impact on nursing / midwifery practice	○	○	○	○
16. An essential role of nurses / midwives is to carry out research	○	○	○	○
17. Most nurses / midwives don’t have any motivation to carry out research	○	○	○	○
18. I think that Nursing / Midwifery research is important	○	○	○	○
19. I think that Nursing / Midwifery research is interesting	○	○	○	○

4. Research Supports

The following questions relate to the degree to which you feel you have the necessary supports and infrastructures to conduct research in your practice.

1. Within your service and / or department how would you rate the following (1= None to 5= Excellent).

	None 1	Low 2	Moderate 3	Good 4	Excellent 5
1 Support given to researchers to undertake clinician research activities	◯	◯	◯	◯	◯
2 Available opportunities in which to participate in sharing research knowledge (inservices, conferences, seminars, forums)	◯	◯	◯	◯	◯
3 The application or use of local research in clinical practice	◯	◯	◯	◯	◯

2. Are research activities considered within your annual performance appraisals?

◯ Yes

◯ No

If yes, what performance indicators are used to assess your research activity?

3. Are research activities considered in your 90 day action plans?

◯ Yes

◯ No

◯ Not applicable

If yes, comment on how it is considered.

4. Please rate your service and / or departments skill or success level for each of the following aspects by rating a score on a scale from 1 to 10 (1=no skill / success and 10=highest possible skill / success)?

	1	2	3	4	5	6	7	8	9	10	Unsure
1. Has adequate resources to support staff research training											
2. Has funds, equipment or admin to support research activities											
3. Does team level planning for research development											
4. Ensures staff involvement in developing that plan											
5. Has team leaders that support research											
6. Provides opportunities to get involved in research											
7. Does planning that is guided by evidence											
8. Has consumer involvement in research activities/planning											
9. Has applied for external funding for research											
10. Conducts research activities relevant to practice											
11. Supports applications for research scholarships/ degrees											
12. Has mechanisms to monitor research quality											
13. Has identified experts accessible for research advice											
14. Disseminates research results at research forums/seminars											
15. Supports a multi-disciplinary approach to research											
16. Has incentives & support for mentoring activities											
17. Has external partners (eg universities) engaged in research											
18. Supports peer-reviewed publication of research											
19. Has software available to support research activities											

5. What are the main clinical practice issues or concerns related to patient care and outcomes that research could potentially have an impact on?

5. Thankyou for your participation

The HNE Nursing and Midwifery Research Centre would like to thank you very much for completing this survey. Your contribution is greatly valued and appreciated.
